# Prediction of axillary lymph node metastasis using a magnetic resonance imaging radiomics model of invasive breast cancer primary tumor

**DOI:** 10.1186/s40644-024-00771-y

**Published:** 2024-09-13

**Authors:** Wei Shi, Yingshi Su, Rui Zhang, Wei Xia, Zhenqiang Lian, Ning Mao, Yanyu Wang, Anqin Zhang, Xin Gao, Yan Zhang

**Affiliations:** 1 Division of Life Sciences and Medicine, School of Biomedical Engineering (Suzhou), University of Science and Technology of China, Suzhou, Jiangsu 215163 China; 2 Medical Imaging Department, Suzhou Institute of Biomedical Engineering and Technology, Chinese Academy of Sciences, Suzhou, Jiangsu 215163 China; 3 Department of Radiology, Guangdong Women and Children Hospital, Guangzhou, Guangdong 511400 China; 4 Department of Radiology, Yantai Yuhuangding Hospital, Qingdao University, Yantai, Shandong 264000 China; 5 Department of Radiology, Zhujiang Hospital, Southern Medical University, Guangzhou, Guangdong 510282 China; 6 Jinan Guoke Medical Engineering and Technology Development Co., Ltd., Jinan, Shandong 250101 China

**Keywords:** Breast cancer primary tumor, Axillary lymph node metastasis, Radiomics

## Abstract

**Background:**

This study investigated the clinical value of breast magnetic resonance imaging (MRI) radiomics for predicting axillary lymph node metastasis (ALNM) and to compare the discriminative abilities of different combinations of MRI sequences.

**Methods:**

This study included 141 patients diagnosed with invasive breast cancer from two centers (center 1: *n* = 101, center 2: *n* = 40). Patients from center 1 were randomly divided into training set and test set 1. Patients from center 2 were assigned to the test set 2. All participants underwent preoperative MRI, and four distinct MRI sequences were obtained. The volume of interest (VOI) of the breast tumor was delineated on the dynamic contrast-enhanced (DCE) postcontrast phase 2 sequence, and the VOIs of other sequences were adjusted when required. Subsequently, radiomics features were extracted from the VOIs using an open-source package. Both single- and multisequence radiomics models were constructed using the logistic regression method in the training set. The area under the receiver operating characteristic curve (AUC), accuracy, sensitivity, specificity, and precision of the radiomics model for the test set 1 and test set 2 were calculated. Finally, the diagnostic performance of each model was compared with the diagnostic level of junior and senior radiologists.

**Results:**

The single-sequence ALNM classifier derived from DCE postcontrast phase 1 had the best performance for both test set 1 (AUC = 0.891) and test set 2 (AUC = 0.619). The best-performing multisequence ALNM classifiers for both test set 1 (AUC = 0.910) and test set 2 (AUC = 0.717) were generated from DCE postcontrast phase 1, T2-weighted imaging, and diffusion-weighted imaging single-sequence ALNM classifiers. Both had a higher diagnostic level than the junior and senior radiologists.

**Conclusions:**

The combination of DCE postcontrast phase 1, T2-weighted imaging, and diffusion-weighted imaging radiomics features had the best performance in predicting ALNM from breast cancer. Our study presents a well-performing and noninvasive tool for ALNM prediction in patients with breast cancer.

## Introduction

Breast cancer is a malignant tumor that poses a threat to women’s health and has become the most prevalent cancer worldwide [1]. The axillary lymph node (ALN) drains approximately 70% of breast lymph, which is the most important lymphatic-transfer pathway in breast cancer. ALN status (with or without metastasis) is an important basis for accurately evaluating clinical stage, treatment strategy, and prognosis in patients with breast cancer [2]. Clinicians commonly perform axillary lymph node dissection to identify ALN status. However, this invasive method is associated with a risk of related complications such as arm numbness and upper limb edema [3]. Therefore, a risk-free method is required for evaluating ALN status, which can reduce unnecessary invasive surgeries and the risk of related complications.

Radiomics, a noninvasive technique, involves resecting a multitude of quantitative features from medical imaging procedures such as computed tomography (CT) scan and magnetic resonance imaging (MRI). These features are subsequently used in lesion diagnosis and the prediction of disease-free survival [4–6]. Notably, radiomics has shown advancements in preoperatively detecting lymph node metastasis in patients with breast cancer. Dong et al. first used radiomics based on fat-suppressed T2-weighted imaging (T2WI) and diffusion-weighted imaging (DWI) MRI sequences to predict ALN status in patients with breast cancer [7]. Their study revealed that the prediction model, based on radiomics features derived from these two sequences, had a high performance with an area under the receiver operating characteristic (ROC) curve (AUC) of 0.805. To enhance the prediction of axillary lymph node metastasis (ALNM), Liu et al. integrated clinicopathological parameters with dynamic contrast-enhanced (DCE) MRI radiomics features [8]. Results showed that the predictive performance of the strategy was comparable to that in the study of Dong et al. In a separate investigation, Chai et al. evaluated the efficacy of predicting ALNM using four different MRI sequences, emphasizing that the second postcontrast phase of DCE had the highest performance, with an AUC of 0.860 [9]. The collective findings underscore the variability in informativeness among MRI sequences for evaluating ALN status. Importantly, incorporating additional MRI sequences into a radiomics model may introduce noise, redundancy, or collinearity among features, potentially compromising model performance, stability, and generalizability [10]. Thus, identifying the optimal combination of MRI sequences is important. Furthermore, the existing prediction models have been commonly developed using data from single centers. Thus, there is a lack of external validation.

This study aimed to explore the optimal combination of multisequence MRI radiomics for developing a prediction model to differentiate the ALN status of patients with breast cancer. Further, the prediction model based on MRI data from an independent center was validated.

## Methods

### Patients and breast MRI acquisition

This study enrolled 101 patients with invasive breast cancer from Guangdong Province Hospital for Women and Children Healthcare (center 1) and 40 patients with invasive breast cancer from Yantai YuHuangDing Hospital (center 2) from March 2021 to January 2022. The inclusion criteria were as follows: (1) patients with histopathological diagnosis of breast cancer, (2) those with information on demographic and clinical characteristics, and (3) those who underwent dedicated breast MRI within 2 weeks before surgery. The exclusion criteria were as follows: (1) patients who received preoperative neoadjuvant chemotherapy or radiotherapy, (2) those with a prior treatment history before MRI, (3) those with other malignant tumors, and (4) those with poor image quality or incomplete sequence.

Using breast tumor tissues, immunohistochemical analysis was performed to determine estrogen receptor (ER), progesterone receptor (PR), and human epidermal growth factor receptor 2 (HER2) status and Ki-67 expression. The molecular subtypes of breast tumors, as per the St. Gallen Consensus Conference 2013, were classified into luminal A (ER + and/or PR+, HER-2−, Ki-67 < 14%), luminal B (ER + and/or PR+, HER-2−, Ki-67 ≥ 14%; ER + and/or PR +, HER-2+, Ki-67 in any state), HER2-positive (ER−, PR−, HER-2+), and triple-negative breast cancer (TNBC, ER−, PR−, HER-2−) [11]. Based on the presence of ALNM, as indicated by the postoperative pathological results, the patients were categorized into the ALNM group and the nonaxillary lymph node metastasis (NALNM) group.

The patients from center 1 underwent imaging using a 3.0T MRI scanner (Ingenia, Philips Healthcare, Best, the Netherlands). Meanwhile, patients from center 2 underwent imaging using a 3.0T MRI scanner (Discovery, GE Healthcare, Milwaukee, Wisconsin, the USA). During the MRI examination, the patients were placed in the prone position. The breasts hang naturally, and they were anchored properly in an eight-channel breast-dedicated coil. In the axial position, the bilateral breasts were in the center of the field of view, including the whole bilateral breasts and axillary region. In the sagittal position, the positioning line was parallel to the long axis of the breast. The imaging protocol comprised non-fat-suppressed T1-weighted imaging (T1WI), fat-suppressed T2WI, DWI, and DCE MRI using fat-suppressed T1WI. DCE had one precontrast phase (1 min before contrast injection) and four postcontrast phases (range: 1–4, corresponding to the first to fourth min after contrast injection). Table 1 shows the details of the MRI protocols.

**Table 1:** MRI protocols of two scanners

Scanner	Sequences	TR/TE	Matrix size	FOV	Slice thickness	Slice spacing
Ingenia 3.0T, Philips Healthcare	T1WI	542/8 ms	272 × 128	450 × 450	4 mm	0.4 mm
	T2WI	4196/75 ms	192 × 221	190 × 339	4 mm	0.4 mm
	DWI (b = 1000 s/mm^2^)	6745/103 ms	212 × 142	400 × 340	4 mm	0.4 mm
	DCE	4.4/2.2 ms	328 × 392	280 × 339	1.6 mm	0.8 mm
Discovery 3.0T, GE Healthcare	T1WI	460/6.3 ms	320 × 140	340 × 340	5 mm	1 mm
	T2WI	5210/85 ms	384 × 384	340 × 340	5 mm	1 mm
	DWI (b = 1000 s/mm^2^)	2496/71 ms	128 × 128	320 × 320	5 mm	1 mm
	DCE	5.7/1.7 ms	288 × 320	360 × 360	2 mm	2 mm

TR: repetition time, TE: echo time, FOV: field of view

### Radiomics feature extraction

**Fig. 1:**
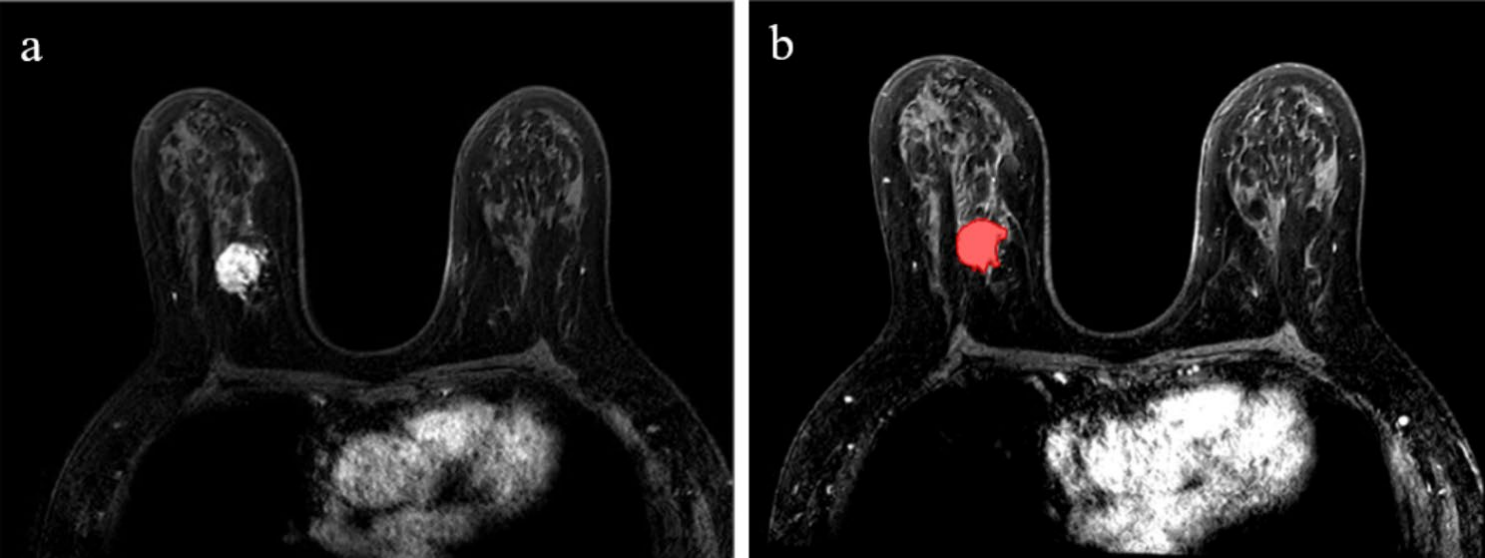
MRI images of a 56-year-old woman with breast cancer. (**a**) DCE postcontrast phase 2 image. (**b**) VOI of the primary tumor manually delineated by the radiologist

A radiologist with 5 years of experience manually delineated the tumor volume of interest (VOI) on DCE postcontrast phase 2 for each patient using the Medical Imaging Interaction Toolkit (MITK) software (v. 2016.11.0; http://www.mitk.org/). Figure 1 shows the VOI of the primary tumor using the MITK. Then, the VOIs of other sequences were manually checked and adjusted if needed.

A series of harmonization techniques were applied to the MRI volumes before radiomics feature extraction [12]. First, images from other sequences were resampled to the resolution, spacing, and position of DCE postcontrast phase 2 using linear interpolation. For each MRI volume, the mean value and the standard deviation of intensity were calculated to standardize the MRI images from all sequences. Next, each volume was normalized using the z-score method by subtracting the mean intensity and dividing by the standard deviation of intensity [13, 14].

PyRadiomics (v3.0.1; http://www.radiomics.io/pyradiomics.html), an open-source Python toolkit for extracting radiomics features from medical images, was used to calculate the radiomics features [15]. In total, 851 radiomics features were calculated from tumor VOI in all sequences. These features included 14 shape features, 18 first-order features, 24 Gy-level cooccurrence matrix features, 16 Gy-level run length matrix features, 16 Gy-level size zone matrix (GLSZM) features, 14 Gy-level dependence matrix features, 5 neighboring gray tone difference matrix features, and 744 Wavelet features. The details of all features are provided online (https://pyradiomics.readthedocs.io/en/latest/features.html) [16].

In the DWI sequence, radiomics features were directly extracted from the diffusion-weighted images. Apparent diffusion coefficient maps for feature extraction were not used.

### Feature selection and ALNM classifier modeling

To reduce irrelevant and redundant features, a three-stage feature selection was performed for each MRI sequence individually. First, the radiomics features with a variance < 0.05 were deleted. Next, Pearson correlation matrixes were established using pair-wise feature correlations. The mean absolute correlation of each feature was calculated, and the one with the highest value was eliminated. Third, to lower the risk of model overfitting, the minimum redundancy maximum relevance (mRMR) feature selection strategy was used to maintain the number of features within 1/10 of the total number of dependent sets [17, 18].

To assess the validity and generality of the ALNM classifier, 101 patients from center 1 were randomly divided into the training set (*n* = 76) and test set 1 (*n* = 25) at a ratio of 3:1. Meanwhile, 40 patients from center 2 were grouped in test set 2 (*n* = 40).

For single-sequence ALNM classifier modeling, the selected radiomics features were fed into the least absolute shrinkage and selection operator (LASSO) [19]. LASSO is a generalized linear model that performs both feature selection and regularization to enhance classification. Moreover, it has a great performance in breast radiomics studies [20]. For each sequence, 3-fold cross-validation was used to achieve a robust single-sequence ALNM classifier. Multisequence ALNM classifiers were constructed by integrating single-sequence ALNM classifiers using the multivariate logistic regression model with combinations of T1WI, T2WI, DWI, and the best-validation-performing DCE phase. In total, four single-sequence ALNM classifiers and 11 multisequence ALNM classifiers were built. The single-sequence ALNM classifiers were the linear weighted sum of radiomics features, and the multisequence ALNM classifiers were the linear weighted sum of the outputs of single-sequence ALNM classifiers.

**Fig. 2:**
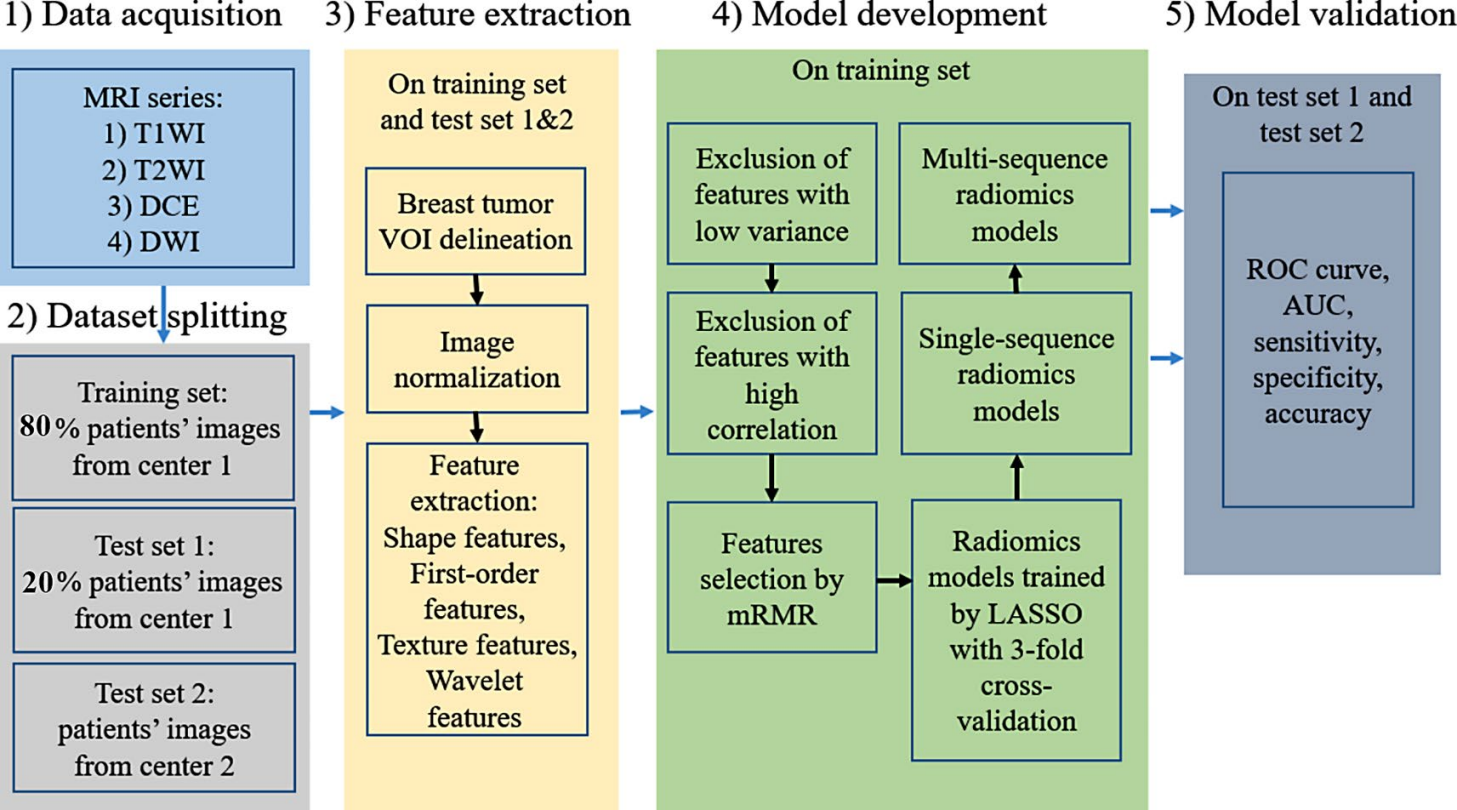
Workflow for the construction and validation of the ALNM classifier

The ROC curve, AUC, sensitivity, specificity, accuracy, and precision of the models were analyzed to assess the performance of the ALNM classifier. Figure 2 shows the flowchart for the ALNM classifier development and validation.

To compare the performance of the radiomics model with the diagnoses of the radiologists, two radiologists (a junior radiologist with 5 years of experience and a senior radiologist with 20 years of experience) were assigned to individually read all original images of each case in test set 1 and test set 2 and to make a diagnostic decision (ALNM vs. NALNM). The ALNM criteria were as follows: enlarged lymph nodes (diameter of ≥ 10 mm) with single or multiple axillary hilar disappearance and uneven circular enhancement on contrast-enhanced MRI images, or small lymph nodes (diameter of < 10 mm) with multiple hilar disappearance and uneven circular enhancement. The NALNM criteria do not fulfill any ALNM criteria.

### Statistical analysis

The categorical variables of patients with ALNM and NALNM were compared using the chi-square test. The Shapiro–Wilk test was used to analyze the distributional properties of continuous variables as mean ± standard deviation for data with a normal distribution or as median (interquartile range) for data with a non-normal distribution. Continuous variables with or without a normal distribution were compared using the Student’s *t*-test or the Wilcoxon rank-sum test, respectively. The differences between ROC curves were compared using the DeLong test [21]. The statistical analysis, feature selection, and development and validation of the model were performed using the R software (version 4.0.3, https://www.r-project.org/). The two-tailed statistical tests were used, and a *P* value of < 0.05 was considered statistically significant.

## Results

### Characteristics of the patients

The age of the patients with NALNM in the training set did not have a normal distribution (*P* = 0.001). There was no significant differences in terms of age, ER, PR, and HER-2 status, Ki-67 expression, and molecular subtypes between the NALNM and ALNM cohorts in the training set, test set 1, and test set 2. Table 2 depicts the clinical and pathological characteristics of the patients.

**Table 2:** Patient profiles in the training set and test set

	Training set (*n* = 76)		*P* value	Test set 1 (*n* = 25)		*P* value	Test set 2 (*n* = 40)		*P* value
**Axillary lymph node status**	Patients with NALNM (*n* = 45)	Patients with ALNM (*n* = 31)		Patients with NALNM (*n* = 16)	Patients with ALNM (*n* = 9)		Patients with NALNM (*n* = 22)	Patients with ALNM (*n* = 18)	
**Age**,** years**									
Median	48		0.096			0.093			0.115
Range	33–76								
Mean ± Std		52.2 ± 9.5		52.4 ± 11.8	59.4 ± 8.02		58.7 ± 6.3	54.3 ± 9.97	
**ER status**									
Negative	14	9	1.000	6	6	0.325	15	17	0.054
Positive	31	22		10	3		7	1	
**PR status**									
Negative	14	11	0.880	6	6	0.325	14	14	0.491
Positive	31	20		10	3		8	4	
**HER-2 status**									
Negative	34	21	0.626	12	6	0.673	4	3	1.000
Positive	11	10		4	3		18	15	
**Ki-67 status**									
Negative	17	5	0.074	3	0	0.280	19	14	0.679
Positive	28	26		13	9		3	4	
**Molecular subtypes**									
Luminal A	15	5	0.189	2	0	0.377	2	4	0.302
Luminal B	17	18		9	3		14	13	
HER-2	5	5		2	3		3	0	
TNBC	8	3		3	3		3	1	

### Feature selection and model validation performance

After excluding features with a variance of < 0.05 and a high correlation, the number of T1WI, T2WI, DWI, DCE precontrast (D_pre), DCE postcontrast phase 1 (D_post_1), DCE postcontrast phase 2, DCE postcontrast phase 3, and DCE postcontrast phase 4 radiomics features was reduced to 236, 209, 236, 189, 185, 180, 187, and 187, respectively. After mRMR and LASSO feature selection, there were 5, 5, 5, 4, 4, 1, 4, and 4 features in the T1WI, T2WI, DWI, DCE precontrast, DCE postcontrast phase 1, DCE postcontrast phase 2, DCE postcontrast phase 3, and DCE postcontrast phase 4 sequences, respectively. Table 3 presents the radiomics features used in single-sequence ALNM classifiers, along with corresponding weights.

**Table 3:** Radiomics features and weights in single-sequence ALNM classifiers

Sequences	Features	Weights
T1WI	(Intercept)	2.775E-01
	original_glszm_LowGrayLevelZoneEmphasis	−5.562E+00
	wavelet.LLH_glszm_SmallAreaLowGrayLevelEmphasis	−1.424E+02
	wavelet.HLL_gldm_SmallDependenceHighGrayLevelEmphasis	−9.228E-03
	wavelet.HHL_firstorder_Minimum	−4.577E-01
	wavelet.HHH_glrlm_LongRunLowGrayLevelEmphasis	3.747E+01
T2WI	(Intercept)	-6.868E+00
	wavelet.LLH_firstorder_90Percentile	5.023E+00
	wavelet.LHL_glrlm_ShortRunHighGrayLevelEmphasis	5.403E-03
	wavelet.HLL_glcm_InverseVariance	8.918E+00
	wavelet.HHH_gldm_DependenceVariance	2.785E-02
	wavelet.LLL_glszm_SmallAreaLowGrayLevelEmphasis	−1.198E+02
DWI	(Intercept)	8.987E-01
	original_glcm_ClusterProminence	−5.022E-06
	wavelet.LLH_glcm_Correlation	1.480E+01
	wavelet.LLH_glcm_MCC	−1.516E+01
	wavelet.LLH_glszm_ZoneEntropy	4.288E-01
	wavelet.HLH_gldm_SmallDependenceHighGrayLevelEmphasis	−1.788E-02
DCE precontrast	(Intercept)	-6.998E+00
	original_gldm_DependenceEntropy	9.370E-01
	wavelet.HLL_firstorder_TotalEnergy	4.213E-05
	wavelet.HLH_glcm_MCC	-2.674E+00
	wavelet.HHH_glrlm_LongRunHighGrayLevelEmphasis	1.601E-03
DCE postcontrast phase 1	(Intercept)	2.145E+00
	original_firstorder_Minimum	−1.004E+00
	wavelet.LLH_gldm_SmallDependenceHighGrayLevelEmphasis	−1.057E-02
	wavelet.HHH_firstorder_Skewness	6.070E+00
	wavelet.LLL_glszm_LowGrayLevelZoneEmphasis	−1.033E+02
DCE postcontrast phase 2	(Intercept)	−4.575E-01
	wavelet.HLH_glcm_ClusterProminence	−8.673E-06
DCE postcontrast phase 3	(Intercept)	2.127E+00
	wavelet.LLH_glcm_Correlation	4.150E+00
	wavelet.LHH_glcm_JointEntropy	−3.072E-01
	wavelet.HLH_glrlm_LongRunLowGrayLevelEmphasis	−5.871E+01
	wavelet.HLH_gldm_SmallDependenceHighGrayLevelEmphasis	−8.902E-03
DCE postcontrast phase 4	(Intercept)	−2.317E+01
	wavelet.LLH_glcm_Correlation	1.713E+00
	wavelet.LHL_gldm_DependenceEntropy	3.524E+00
	wavelet.HLL_glrlm_ShortRunHighGrayLevelEmphasis	1.039E-03
	wavelet.HHH_gldm_SmallDependenceHighGrayLevelEmphasis	−8.965E-03

**Fig. 3:**
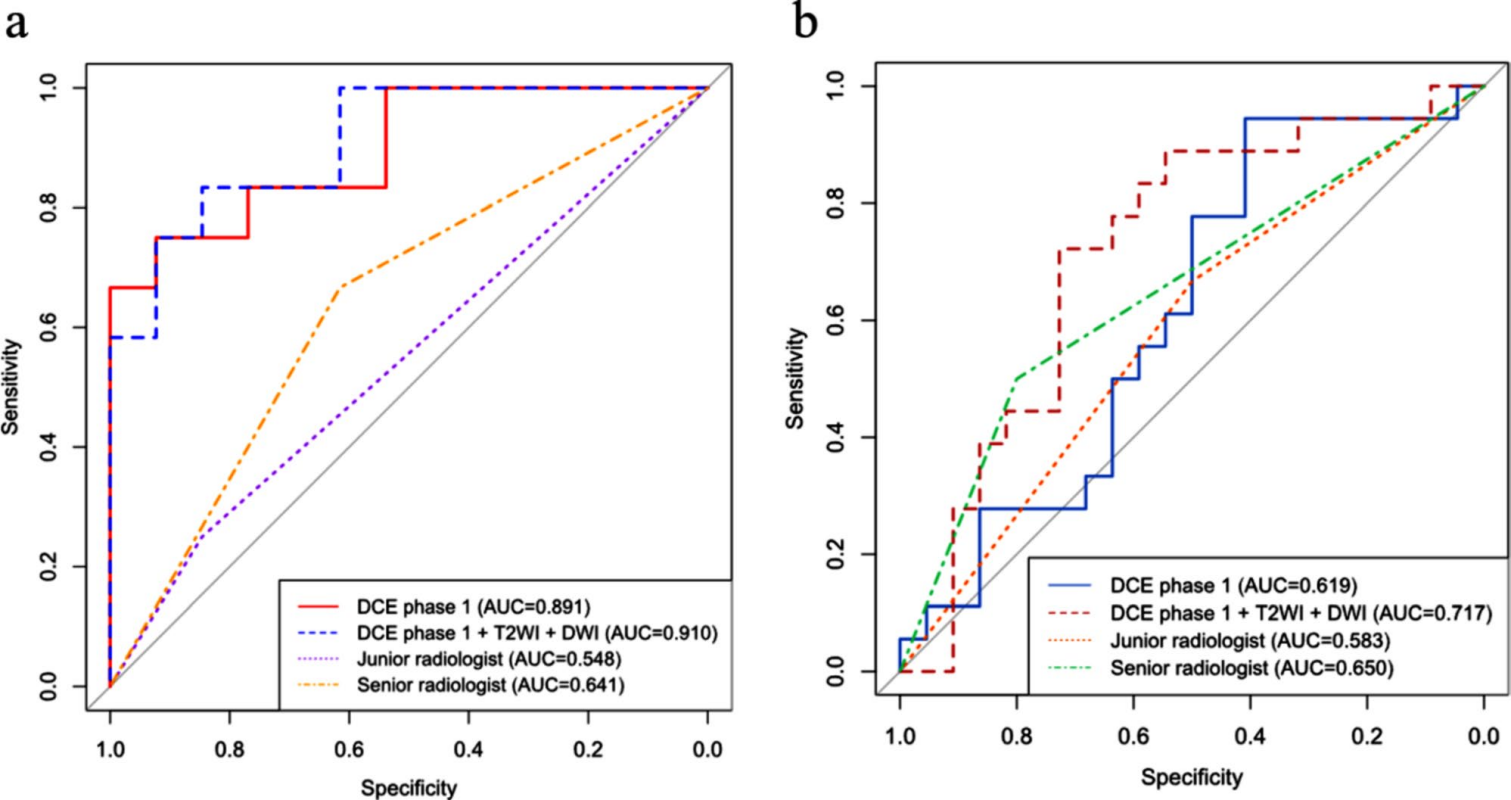
ROC curves of the ALNM classifiers and two radiologists. (**a**) Test set 1, (**b**) Test set 2

**Fig. 4:**
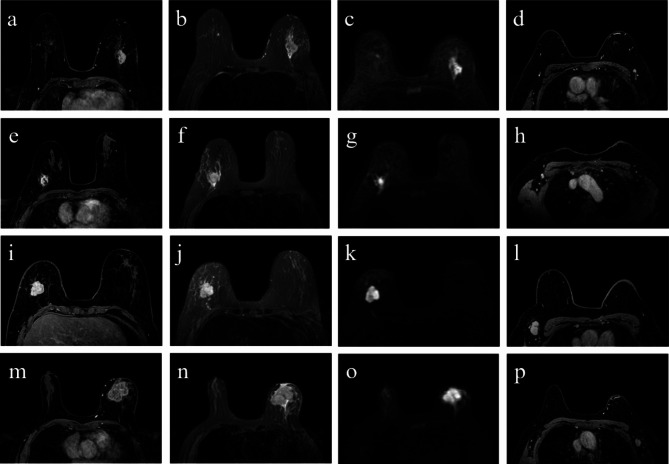
MRI images of four representative cases. (**a**, **e**, **i**, and **m**) DCE postcontrast phase 1 images of the primary tumor. (**b**, **f**, **j**, and **n**) T2WI images of the primary tumor. (**c**, **g**, **k**, and **o**) DWI images of the primary tumor. (**d**, **h**, **l**, and **p**) DCE postcontrast phase 1 images of ALN. (**a**–**d**) A 57-year-old female patient with breast cancer presented with pathologically confirmed left ALNM. The patient was misdiagnosed with NALNM by the junior radiologist but was correctly diagnosed by the senior radiologist and the combined DCE postcontrast phase I + T2WI + DWI model. (**e**–**h**) A 66-year-old female patient with breast cancer presented with pathologically confirmed right NALNM. The patient was misdiagnosed with ALNM by the senior radiologist but was correctly diagnosed by the junior radiologist and the combined DCE postcontrast phase I + T2WI + DWI model. (**i**–**l**) A 65-year-old female patient with breast cancer presented with pathologically confirmed right NALNM. The patient was misdiagnosed with ALNM by the senior and junior radiologists but was correctly diagnosed by the combined DCE postcontrast phase I + T2WI + DWI model. (**m**–**p**) A 59-year-old female patient with breast cancer who presented with pathologically confirmed left ALNM. The patient was correctly diagnosed by the senior and junior radiologists and the combined DCE postcontrast phase I + T2WI + DWI model

Table 4 presents the AUC, accuracy, sensitivity, and specificity of single-sequence and multisequence ALNM classifiers. The single-sequence ALNM classifier derived from DCE postcontrast phase 1 had the best performance for both test set 1 (AUC = 0.891) and test set 2 (AUC = 0.619) among all DCE phases. The best-performing multisequence ALNM classifier for both test set 1 (AUC = 0.910) and test set 2 (AUC = 0.717) was generated from DCE postcontrast phase 1, T2WI, and DWI single-sequence ALNM classifiers. In test set 1, the AUC of the DCE postcontrast phase 1 + T2WI + DWI model was significantly higher than that of the senior radiologist (0.910 vs. 0.641, *P* = 0.012). In test set 2, the AUC of the DCE postcontrast phase 1 + T2WI + DWI model did not significantly differ from that of the senior radiologist (0.717 vs. 0.650, *P* = 0.569). Figure 3 shows the ROC curves of the best-performing single-sequence, multisequence ALNM classifiers, and two radiologists. Figure 4 depicts the MRI images of four representative cases.

**Table 4:** Performance of the ALNM models and radiologists

Sequences	Test set 1					Test set 2				
	AUC	ACC	SEN	SPE	Prec	AUC	ACC	SEN	SPE	Prec
T1	0.821	0.800	0.833	0.769	0.833	0.593	0.625	0.778	0.500	0.538
T2	0.788	0.760	1.000	0.538	0.667	0.712	0.725	0.667	0.773	0.706
DWI	0.782	0.800	0.583	1.000	0.875	0.591	0.650	0.500	0.773	0.778
DCE Precontrast	0.865	0.880	1.000	0.769	0.800	0.551	0.575	0.722	0.455	0.400
DCE1	0.891	0.840	0.750	0.923	0.900	0.619	0.650	0.944	0.409	0.567
DCE2	0.846	0.800	0.833	0.769	0.714	0.591	0.575	1.000	0.227	0.529
DCE3	0.821	0.800	0.917	0.692	0.667	0.558	0.650	0.611	0.682	0.579
DCE4	0.724	0.720	1.000	0.462	0.692	0.545	0.575	0.889	0.318	0.514
DCE1 + T1	0.853	0.840	0.917	0.769	0.786	0.624	0.650	0.833	0.500	0.577
DCE1 + T2	0.885	0.880	0.917	0.846	0.846	0.702	0.725	0.889	0.591	0.640
DCE1 + DWI	0.897	0.840	0.750	0.923	0.900	0.624	0.675	1.000	0.409	0.581
T1 + T2	0.885	0.880	0.833	0.923	0.909	0.667	0.675	0.667	0.682	0.632
T1 + DWI	0.897	0.840	0.833	0.846	0.833	0.598	0.575	0.889	0.318	0.516
T2 + DWI	0.904	0.840	1.000	0.692	0.750	0.712	0.725	0.722	0.727	0.684
DCE1 + T1 + T2	0.891	0.880	0.833	0.923	0.909	0.669	0.700	0.611	0.773	0.688
DCE1 + T1 + DWI	0.904	0.840	0.750	0.923	0.900	0.631	0.650	0.833	0.500	0.577
DCE1 + T2 + DWI	0.910	0.840	0.833	0.846	0.833	0.717	0.725	0.722	0.727	0.684
T1 + T2 + DWI	0.885	0.840	0.833	0.846	0.833	0.669	0.675	0.667	0.682	0.632
DCE1 + T1 + T2 + DWI	0.891	0.840	0.833	0.846	0.833	0.672	0.675	0.667	0.682	0.632
Junior radiologist	0.548	0.560	0.250	0.846	0.600	0.583	0.375	0.333	0.500	0.667
Senior radiologist	0.641	0.640	0.667	0.615	0.615	0.650	0.425	0.500	0.200	0.652

AUC: area under the receiver operating characteristic curve, ACC: accuracy, SEN: sensitivity, SPE: specificity, Prec: precision, T1: T1-weighted imaging, T2: T2-weighted imaging, DCE1: dynamic contrast-enhanced postcontrast phase 1, DCE2: dynamic contrast-enhanced postcontrast phase 2, DCE3: dynamic contrast-enhanced postcontrast phase 3, DCE4: dynamic contrast-enhanced postcontrast phase 4

## Discussion

This retrospective study compared the performance of prediction models based on different combinations of multisequence MRI radiomics features for differentiating the ALN status of patients with breast cancer. The models were developed using data from two distinct centers, enhancing their generalizability and robustness, particularly with the inclusion of data from the second center as an external validation set. The model incorporating the radiomics features from DCE postcontrast phase 1, T2WI, and DWI had the best performance in both the internal and external validation sets. Furthermore, the diagnostic performance between the MRI radiomics model and two radiologists was performed. Results showed that the MRI radiomics model had a better diagnostic efficiency than radiologists.

Our findings are in accordance with a subset of prior studies showing that clinicopathologic features, including age, ER, PR, and Her-2 status, and Ki-67 expression, and molecular subtypes were not significantly correlated with ALNM outcomes [20, 22, 23]. Consequently, these clinicopathologic characteristics were excluded from our models. Nonetheless, the prevailing view of researchers is that ER and PR status, ki-67 expression, and molecular types can be predictive factors of ALNM [24–28]. The differences may be attributed to the inclusion of various study populations, use of a small sample size, and uneven distribution of the sample size in our study.

The single-sequence ALNM classifier derived from DCE postcontrast phase 1 had the best performance in the test set 1 (AUC = 0.891) and test set 2 (AUC = 0.619). This result is similar to that of Liu et al. [29]. This may be because DCE-MRI can diagnose breast diseases by evaluating tumor morphology and hemodynamics. Thus far, breast cancer is a common type of tumor with a rich blood supply, and enhancement is more pronounced in the early stage. Therefore, DCE postcontrast phase 1 is the most effective in displaying the boundaries, heterogeneity, and invasiveness of breast cancer lesions [30].

Numerous studies have investigated the optimal combination of sequences, often selecting T2WI, DWI, and enhancement sequences based on priori experiences [7, 8]. Dong et al. reported that the AUC of a radiomics model combining T2WI and DWI was 0.805 [7]. DWI images can provide additional insights into the diffusion–perfusion characteristics of the primary tumor [31]. T2WI is known for its superior tissue contrast, offering textural features that enhance discrimination. In our study, the inclusion of DCE postcontrast phase 1 radiomics and T2WI and DWI radiomics resulted in a higher AUC (0.910 in test set 1). Therefore, the enhancement sequence improves lesion visualization, which is in accordance with clinical observations. Our study showed that the optimal multisequence ALNM classifier outperformed the junior and senior radiologists in terms of AUC scores (0.910 vs. 0.641 vs. 0.548 in test set 1 and 0.717 vs. 0.583 vs. 0.650 in test set 2). This underscores the utility of radiomics models in clinical diagnostics, a conclusion supported by established research [32, 33].

The single-sequence ALNM classifier derived from DCE postcontrast phase 1 comprised features from the First_order features and GLSZM measures. This finding is consistent with that of previous radiomics studies [34, 35]. In the T2WI- and DWI-based model, the wavelet features were the predominant features, which is consistent with the study showing that wavelet features must be the building blocks of radiomics models [36].

Similar to previous studies, our study predicted the status of ALNM based solely on radiomics features extracted from the ROIs of primary tumors. Radiomics models incorporating ROIs from the peritumor region can possibly provide a richer texture information than those based solely on primary tumor ROIs, thereby improving the accuracy and completeness of prediction models [37]. For example, Liu et al. established radiomics models using intratumoral, 3-mm peritumoral, and 5-mm peritumoral radiomics features from DCE-MRI to predict ALNM status using various machine learning algorithms [38]. Their results indicated that the combined intratumoral and 3-mm peritumoral model, constructed using the BPNN algorithm, exhibited the best predictive performance. Hence, the tumor peripheral microenvironment can play a significant role in predicting tumor aggressiveness.

Recent advancements in abbreviated (AB)-MRI have gained attention due to their potential to reduce MRI costs by shortening image acquisition and interpretation times [39]. A meta-analysis assessing the diagnostic accuracy of AB-MRI against full diagnostic protocol MRI (FDP-MRI) in both the screening and enrichment cohorts found no significant differences in terms of sensitivity or specificity between the two methods [40]. Several AB-MRI protocols recommend the inclusion of T1-weighted pre- and postcontrast sequences. This study identified an optimal sequence combination of T2WI, DCE postcontrast phase 1, and DWI, which not only supports the practicality of AB-MRI but also offers innovative directions for its protocols. Notably, in the AB-MRI protocol proposed by Kuhl et al. [41], in addition to the conventional sequences including DCE precontrast and postcontrast phase 1, there were also special reconstruction sequences such as subtraction and maximum-intensity projection (MIP). Results showed that the diagnostic performance of the AB-MRI protocol was comparable to that of the full diagnostic protocol. In our retrospective study, due to the limited storage capacity of the PACS system, the subtraction, MIP sequences of each patient could not be obtained in time, and only the normal reconstruction sequences were used. We can add MIP and subtraction sequences purposely in the future and continually search for the combination of MRI sequences with a higher diagnostic efficacy. Moreover, breast MRI is advantageous for evaluating axillary lymphatic conditions. However, mammography and contrast-enhanced spectral mammography do not comprehensively cover axillary lymph nodes. This study introduces a novel approach for predicting ALNM by delineating original lesions on mammography or contrast-enhanced spectral mammography images, a method validated by the findings of Mao et al. [42].

### Limitations

This study had some limitations. First, although our findings are promising, the limited number of patients affect the generalizability of our results. Thus, we plan to expand our study by including more patients and collaborating with additional centers. Second, the heterogeneity of images from different machines in multiple centers could not be prevented. Third, radiomics feature extraction required the presegmentation of VOIs, which is still dependent on manual delineation by radiologists. This step is time-consuming, at a risk of error, and has a low reproducibility. If imaging histology modeling is applied in clinical practice, a reliable and efficient automated segmentation method should be identified [43, 44]. Moreover, our study only included MRI radiomics features and clinicopathologic features. The model did not use ALNs and peritumor radiomics features, which limited the comprehensiveness of the prediction model. Finally, DCE-MRI images for VOIs were used exclusively. DCE images offer valuable temporal information about the “wash-in” and “wash-out” of contrast agents. However, they may not fully leverage the enhanced contrast available in the difference images between postcontrast and precontrast phases. Difference images can provide a stronger contrast, potentially leading to a more precise tumor delineation.

## Conclusion

The impact of several MRI sequences on ALNM prediction was examined. This comparative study is beneficial to the community because it can provide a better comprehension of the relative benefits of various MRI sequences on radiomics based ALNM distinction. Nevertheless, future studies should enroll patients from different centers, include a larger sample size, and develop more reliable predictive models with a greater generalization ability.

## Data Availability

The datasets generated and/or analyzed in the current study are available from the corresponding author upon reasonable request.
